# Rapid Development of an Injection Mold with High Cooling Performance Using Molding Simulation and Rapid Tooling Technology

**DOI:** 10.3390/mi12030311

**Published:** 2021-03-16

**Authors:** Chil-Chyuan Kuo, Trong-Duc Nguyen, Yi-Jun Zhu, Shi-Xun Lin

**Affiliations:** 1 Department of Mechanical Engineering, Ming Chi University of Technology, No. 84, Gungjuan Road, New Taipei City 243303, Taiwan; M05118016@mail.mcut.edu.tw (T.-D.N.); M09118003@mail.mcut.edu.tw (Y.-J.Z.); U06117018@mail.mcut.edu.tw (S.-X.L.); 2 Research Center for Intelligent Medical Devices, Ming Chi University of Technology, No. 84, Gungjuan Road, New Taipei City 243303, Taiwan

**Keywords:** conformal cooling channels, rapid tooling technology, cooling time

## Abstract

Rapid tooling technology (RTT) provides an alternative approach to quickly provide wax injection molds for the required products since it can reduce the time to market compared with conventional machining approaches. Removing conformal cooling channels (CCCs) is the key technology for manufacturing injection mold fabricated by rapid tooling technology. In this study, three different kinds of materials were used to fabricate CCCs embedded in the injection mold. This work explores a technology for rapid development of injection mold with high cooling performance. It was found that wax is the most suitable material for making CCCs. An innovative method for fabricating a large intermediary mold with both high load and supporting capacities for manufacturing a large rapid tooling using polyurethane foam was demonstrated. A trend equation for predicting the usage amount of polyurethane foam was proposed. The production cost savings of about 50% can be obtained. An optimum conformal cooling channel design obtained by simulation is proposed. Three injection molds with different cooling channels for injection molding were fabricated by RTT. Reductions in the cooling time by about 89% was obtained. The variation of the results between the experiment and the simulation was investigated and analyzed.

## 1. Introduction

Sustainability as well as cost and time are key issues in the development of an injection mold with conformal cooling channels (CCCs). Injection molding is a manufacturing process for manufacturing parts by injecting material into a mold [1,2]. The CCCs have a uniform distance between the mold surfaces and the channels so that the cooling effect is better than conventional cooling channels. Both final part quality and cycle time can be improved by the mold with the CCCs. Conformal cooling donates the cooling channels that conform to a part’s geometry. However, traditional milling and drilling methods cannot produce intricate CCCs. Additive manufacturing (AM) processes such as fused filament fabrication (FFF), electron beam melting, selective laser melting [3], selective laser sintering [4], diffusion bonding [5], direct metal deposition [6], or direct metal laser sintering [7] overcome these challenges and enable the production of molds or dies with complex CCCs. Kitayama et al. [8] examined the cooling performance of CCCs in plastic injection molding (PIM) numerically and experimentally. Results showed that the CCCs provide reduction on the warpage of the injection molded part. Holker and Tekkaya [9] developed extrusion dies with conformal cooling channels for increasing the productivity in hot aluminum extrusion. It was found that remarkable increases in productivity can be achieved by applying the proposed tooling technology in hot extrusion. Lim et al. [10] proposed a method for designing the cooling channel by means of the energy balance principle and arrangement method. The results clearly showed that both hardness and tensile strengths of the products were improved. Wang et al. [11] employed optimization of mold with spiral conformal cooling system and product structure to reduce service stress of the injection molded parts. It was found that the common injection molding defects such as residual stress and warpage cannot be ignored. Brooks and Brigden [12] proposed a concept for designing the conformal cooling layers with self-supporting lattices for additively manufactured tooling. This work showed that the cooling time of the injection molded part can be reduced approximately 26% when the conformal cooling layers were utilized. Vojnova [13] introduced the benefits of molds with conformal cooling systems in the injection molding process. The results clearly demonstrated that the CCCs are suitable for geometrically complex molds to remove the heat generated in areas where this is not possible with conventional methods. It is well known that rapid tooling technology (RTT) can produce a mold economically and efficiently compared with conventional methods. A rapid tool with different cross-sectional cooling channels was developed [14,15]. A reduction in cooling time of about 81% was obtained. In addition, an injection mold fabricated by RTT had some distinct advantages, including mold size, interior quality of the CCCs, production costs, and post-processing operations compared with injection mold fabricated by metal AM, such as selective laser sintering (SLS), selective laser melting (SLM), and diffusion bonding (DB) technologies. A low-cost wax injection mold with high cooling efficiency was developed [16,17]. A hot embossing stamp with CCCs for microreplication was developed [18]. Results showed that the reduction in cooling time of about 92% was obtained when a hot embossing stamp had conformal cooling channels compared to the hot embossing stamp, which has conventional cooling channels. In addition, the production cost reduction of a hot embossing stamp with microstructure of approximately 72% was obtained using RTT.

Based on experience of making injection mold with complex CCCs, removing CCCs from the injection mold fabricated by rapid tooling technology is a very complicated and difficult process. In addition, poorly designed cooling channels will reduce the cooling efficiency of the injection mold. The core manufacturing technology for rapid development of injection mold with optimum CCCs was demonstrated. Simulation software [19] was used to optimize CCCs embedded in the injection mold. A high cooling efficiency injection mold with the optimum design of conformal cooling channels was fabricated by RTT. The cooling time and characteristics of the injection mold was investigated experimentally. The cooling performance of the injection molds with and without cooling channels was compared experimentally.

## 2. Experimental Details

**Figure 1:**
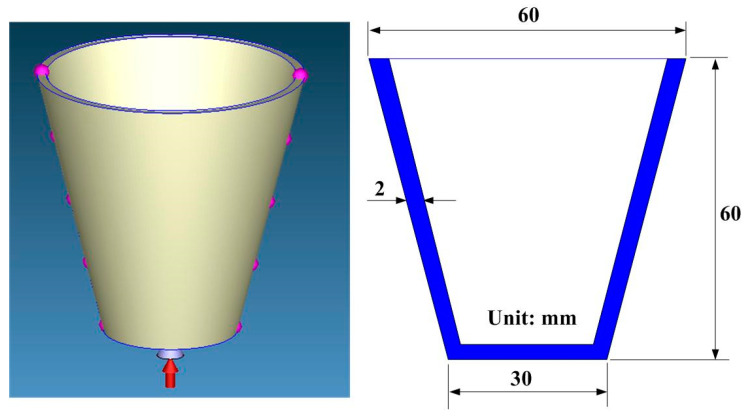
CAD model and cross section of the injection molded part.

**Figure 2:**
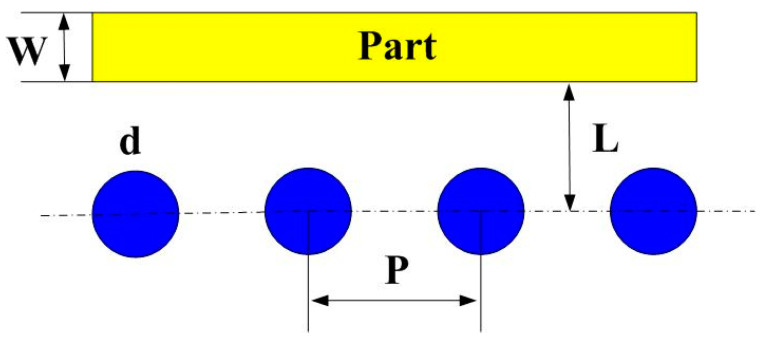
Schematic illustrations of the four cooling channel design parameters.

The master model, cooling channels, and injection molds were designed using SolidWorks software. Figure 1 shows the computer-aided design (CAD) model and cross section of the injection molded part. The master model is a water cup with the dimensions of 60 mm in upper outer diameter, 30 mm in lower outer diameter, 60 mm in height, and 2 mm in wall thickness. The volume of the master model is 17.3 cc. The main reason for choosing the water cup as an injection molded product is that the top edge of the water cup is set as a parting surface, which can easily disassemble the core and cavity inserts. Moreover, the CCCs geometries designed for core and cavity insert do not overlap. Thus, choosing the water cup as an injection molded product can demonstrate the substantial benefits of rapid injection tool with CCCs. The main factors affecting the cooling efficiency of the conformal cooling channels include the diameter of the cooling channels, center distance with respect to the mold cavity, and center distance between cooling channels. Figure 2 shows the schematic illustrations of the four cooling channel design parameters [20,21]. The W, d, P, and L stand for wall thickness of the injection molded part, cooling channel diameter, center distance between cooling channels, and center distance with respect to mold cavity, respectively. There are three different methods for designing conformal cooling channels, i.e., spiral, zigzag, and parallel [22]. It should be noted that the spiral conformal cooling channels were used in this study.

**Figure 3:**
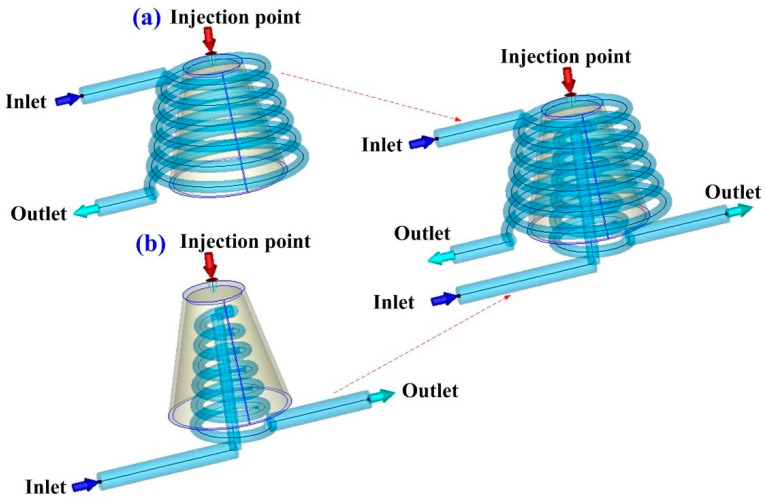
Schematic illustration of the injection molded part with conformal cooling channels of (**a**) cavity insert and (**b**) core insert.

**Figure 4:**
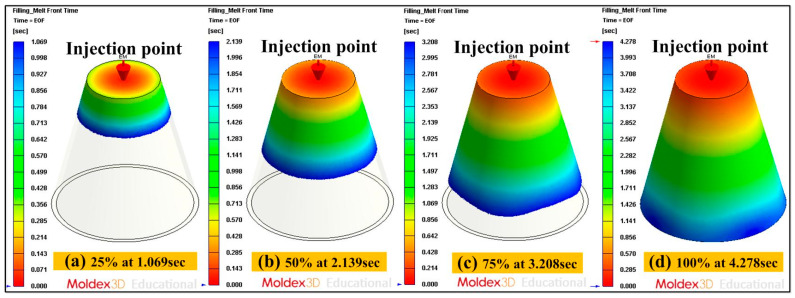
Filling results of the injection molded partfor filling ratio (**a**) 25%, (**b**) 50%, (**c**) 75% and (**d**) 100%.

According to conformal cooling channel design guidelines, the cooling channel diameter was about 4 to 8 mm because the wall thickness of the injection molded part was 2 mm. The center distance was about 1.5 to 2 times that of the cooling channel diameter. Thus, the center distance between cooling channels was set at 6 to 16 mm. The center distance with respect to mold cavity was about 2 to 3 times of cooling channel diameter. Thus, the center distance with respect to mold cavity was set 8 to 24 mm theoretically. The five different cooling channel diameters were used in this study. Each diameter of the cooling channel matched with three different center distances between cooling channels and center distances with respect to mold cavity. Three different center distances between cooling channels were 1.5, 1.7, and 2 times the cooling channel diameters, respectively. Three different center distances with respect to mold cavity were 2, 2.5, and 3 times the cooling channel diameters, respectively. Therefore, forty-five cases of conformal cooling channel design were carried out for experiment, as shown in Table 1. The Moldex3D simulation software (R14 SP3OR, CoreTech System Inc., Hsinchu, Taiwan) was used to investigate the optimum CCCs. Injection molds with different layout of cooling systems were fabricated with aluminum (Al)-filled epoxy resin (TE-375, Jasdi Inc., Taoyuan, Taiwan) by RTT. Table 2 gives the boundary and initial conditions for numerical simulation. The commercially available wax (K512, Kato Inc., Taoyuan, Taiwan) was used for fabrication of for CCC fabrication using a low-pressure wax injection molding machine (0660, W&W Inc., Taoyuan, Taiwan). The process parameters for injection molding were injection pressure of 0.18 MPa, injection time of 4.3 s, and injection temperature of 82 °C. Figure 3 shows the schematic illustration of the injection molded part with conformal cooling channels of both cavity insert and core insert. The filling time of the injection molded part was approximately 4.278 s, as shown in the Figure 4. As can be seen, the injection molded part can be filled completely under the steady-state process settings. In general, the pressure drop of the sprue gate was very small because the molded materials can flow directly into the mold cavity without passing through the intricate runner system. Therefore, a sprue gate was selected.

**Figure 5:**
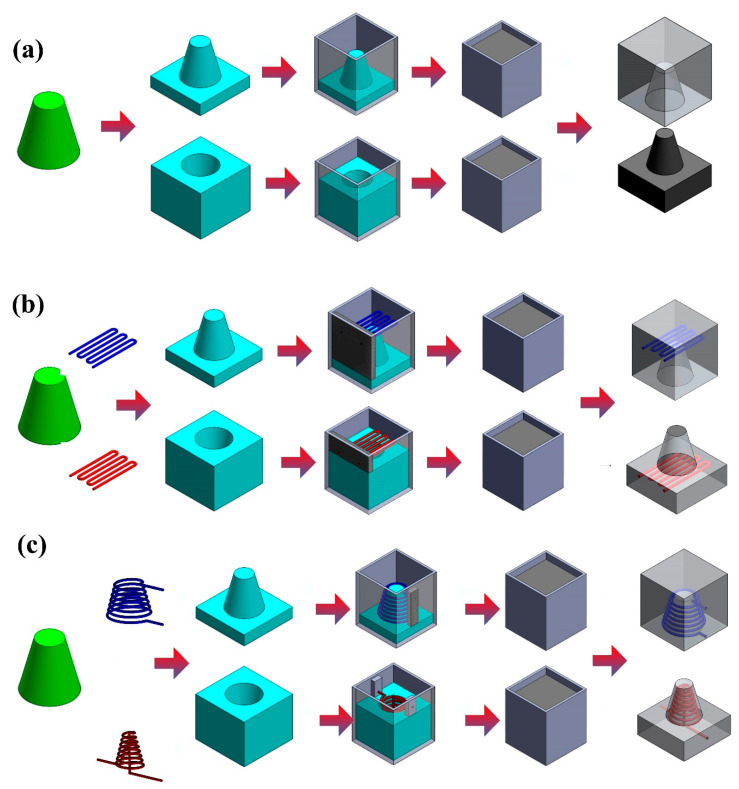
Process layouts for fabricating three different kinds of injection molds (**a**) without cooling channels, (**b**) with conventional cooling channels, and (**c**) with conformal cooling channels.

The master model was fabricated using a FFF system (YK-210, Youkung Inc., New Taipei City, Taiwan) through polylactic acid (PLA) plastics with a layer thickness of 0.254 mm. Support materials of the master model were removed using alkaline detergent solution. The pH meter (pH 600) was used to measure the pH value of the solution before removing support materials. The injection mold fabricated only had one mold cavity. The AM was used to fabricate CCCs through three different materials such as acrylonitrile butadiene styrene (ABS), PLA plastics, and wax since it provides the flexibility in the fabrication of CCCs compared to conventional machining technologies. The process parameters for making PLA CCCs were printing temperature of 200 °C, hot bed temperature of 50 °C, printing speed of 50 mm/s, and layer thickness of 0.2 mm. The process parameters for making ABS CCCs were printing temperature of 300 °C, hot bed temperature of 100 °C, printing speed of 50 mm/s, and layer thickness of 0.2 mm. The process parameters for making wax CCCs were printing temperature of 140 °C, hot bed temperature of 50 °C, printing speed of 20 mm/s, and layer thickness of 0.2 mm. Figure 5 shows the process layouts for fabricating three different kinds of injection molds. An intermediary mold that was complementary in shape to the injection mold was first fabricated through the master model using silicone rubber. To reduce the production cost of a large intermediary mold, an innovative method using polyurethane foam (PUF) was proposed. A vacuum machine (F-600, Feiling Inc., Taoyuan, Taiwan) was used to eliminate air bubbles from the resulting mixture. The injection molds were then fabricated through an intermediary tooling using the Al-filled epoxy resins. Finally, the injection molds fabricated were cured using a convection oven (DH400, Deng Yag Inc., Taoyuan, Taiwan) at 150 °C for 2 h to obtain the mechanical properties required for injection molding.

**Figure 6:**
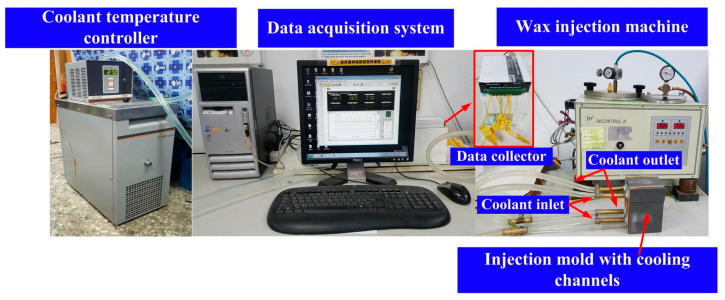
The layout of the experimental setup for investigating the cooling efficiency of the fabricated injection molds.

**Figure 7:**
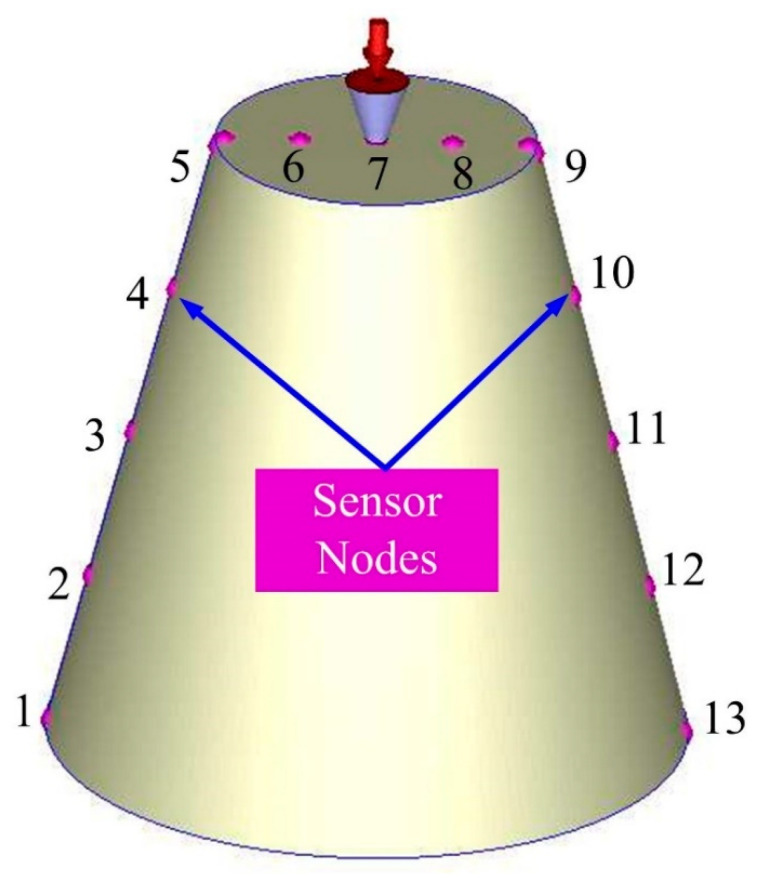
Locations of sensor nodes.

To evaluate the cooling performance of the injection molds with different layouts of cooling systems, the layout of the experimental setup is shown in the Figure 6. This system was composed of a thermo-electric cooler (TEC12706AJ, Caijia Inc., Taoyuan, Taiwan) and a temperature controller (JCM-33A, Shinko Inc., Taoyuan, Taiwan). The inlet coolant temperature was kept at room temperature. Three k-type thermocouples (C071009-079, Cheng Tay Inc., New Taipei City, Taiwan) were embedded in the injection molds for on-line recording temperature history of the injection molded part, inlet coolant temperature, and outlet coolant. Temperature data were recorded by a data acquisition system (MRD-8002L, IDEA System Inc., Taoyuan, Taiwan). To increase the cooling efficiency, the flow of coolant with Reynolds number more than 10,000 was performed by the water pump. The ejection temperature of the injection molded parts was set at room temperature. The cooling time of the fabricated three injection molds with different types of cooling channels after the wax injection molding was measured and analyzed. To ensure the temperature distribution across the whole injection molded part, 13 temperature sensor nodes were carried out. Figure 7 shows the locations of sensor nodes.

## 3. Results and Discussion

**Figure 8:**
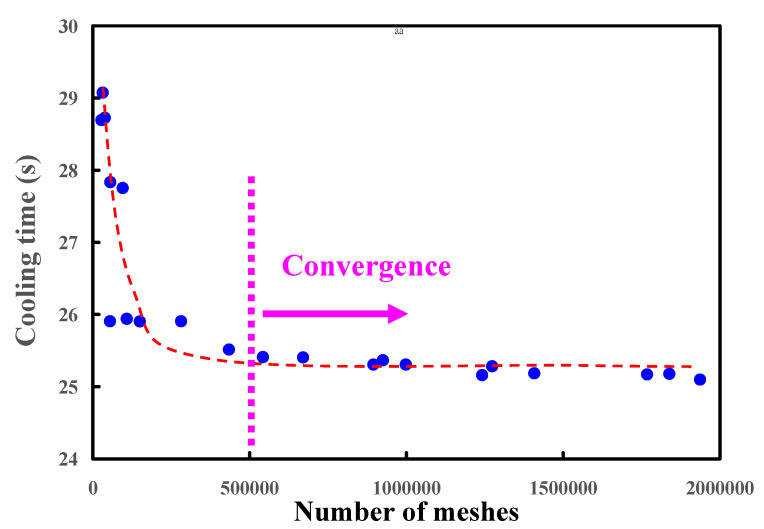
Convergence study of the number of meshes.

**Figure 9:**
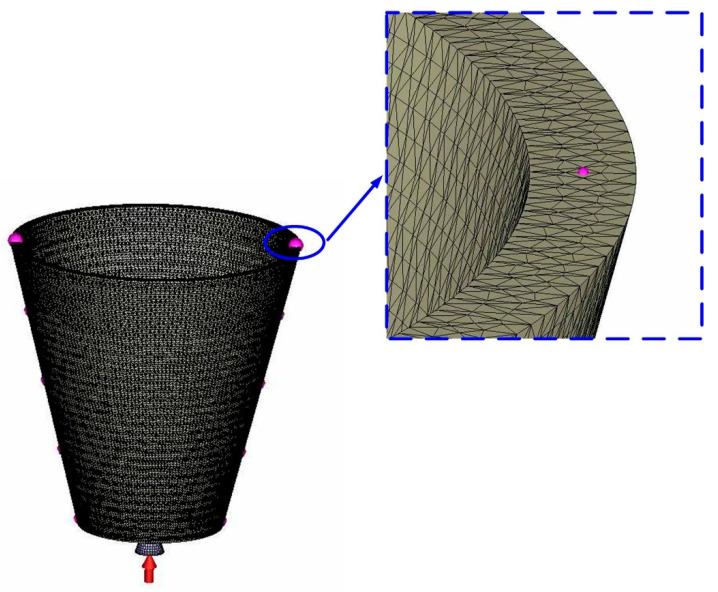
Mesh model of the molded product.

**Figure 10:**
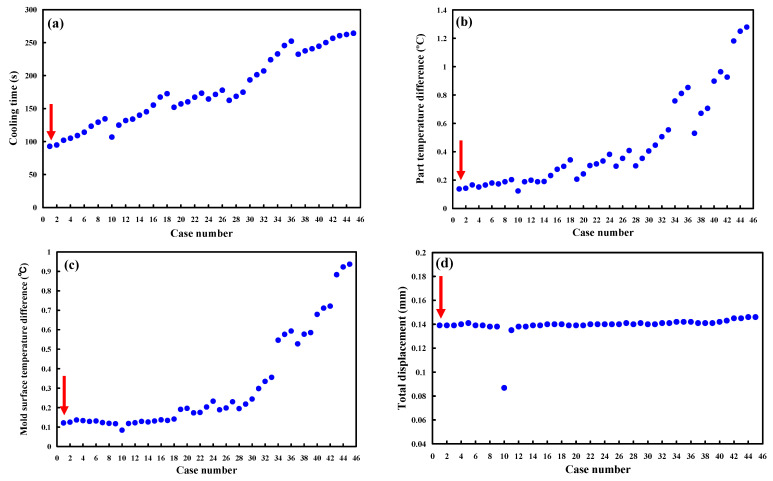
Simulation results of the forty-five cases on the (**a**) cooling time, (**b**) part temperature difference, (**c**) mold surface temperature difference, and (**d**) total displacement.

The 3D simulation models were imported from CAD software to Moldex 3D simulation software through a data exchange STEP format. The 3D solid mesh included four kinds of meshes, including tetra, pyramid, hexahedron, and prism. The number of nodes for tetra, pyramid, and hexahedron were 4, 5, and 6, respectively. To ensure accuracy of mold-filling analyses, boundary layer mesh was used since it is suitable for simulation models with complex geometries. The simulation models were composed of tetrahedron, pyramid, and hexahedron meshes. Figure 8 shows the convergence study of the number of meshes. This result reveals that the cooling time will converge after the number of mesh elements exceeds 500,000. Figure 9 shows the mesh model of the molded product. The mesh node counts and the mesh element counts were 791,548 and 1,260,635, respectively. Figure 10 shows the simulation results of the forty-five cases on the cooling time, part temperature difference, mold surface temperature difference, and total displacement. Table 3 shows the simulation results of the forty-five cases. Figure 10a shows the simulation results of the cooling time. It was found that the lowest values of the cooling time, part temperature difference, mold surface temperature difference, and total displacement were 92.73 s, 0.123 °C, 0.084 °C, and 0.086 mm, respectively. The average values of the cooling time, part temperature difference, mold surface temperature difference, and total displacement were 175.9 s, 0.439 °C, 0.31 °C, and 0.14 mm, respectively. From these results, the benefits of the Moldex3D simulation can be clearly found. As can be seen, the cooling time was reduced by 83.17 s compared with the average values. The part temperature difference was reduced by 0.32 °C compared with the average values. The mold surface temperature difference was reduced by 0.226 °C compared with the average values. The total displacement was reduced by 0.054 °C compared with the average values. It was remarkable that the benefits using Moldex3D simulation in the cooling time, part temperature difference, mold surface temperature difference, and product total deformation were 47.28, 72.01, 72.90, and 38.22%, respectively. The cooling channel diameter, center distance between cooling channels, and center distance with respect to mold cavity for the case number 1 were 4, 6, and 8 mm, respectively. The cooling channel diameter, center distance between cooling channels, and center distance with respect to mold cavity for the case number 10 were 5, 7.5, and 10 mm, respectively. For the conformal cooling design 1, the cooling time, part temperature difference, mold surface temperature difference, and total displacement were 92.73 s, 0.137 °C, 0.121 °C, and 0.139 mm, respectively. For the conformal cooling design 10, the cooling time, part temperature difference, mold surface temperature difference, and total displacement were 106.8 s, 0.123 °C, 0.084 °C, and 0.086 mm, respectively. Figure 10b shows the simulation results of the part temperature difference. Figure 10c shows the simulation results of the mold surface temperature difference. Figure 10d shows the simulation results of the total displacement. The part temperature difference, mold surface temperature difference, and total displacement were slightly increased by 0.014 °C, 0.037 °C, and 0.053 mm when the results of the conformal cooling design 1 was compared with that of the conformal cooling design 10. In contrast, the cooling time was significantly reduced by 14.07 s. In general, the cooling time was related to the production rate. Three parameters including part temperature difference, mold surface temperature difference, and total displacement were related to the molded part quality. It was concluded that the case number one seems can be recommended as an optimum design of conformal cooling channels in terms of the productivity and product quality. Thus, an optimum conformal cooling channel design was that the cooling channel, center distance between cooling channels, and center distance with respect to mold cavity were 4, 6, and 8 mm, respectively.

**Figure 11:**
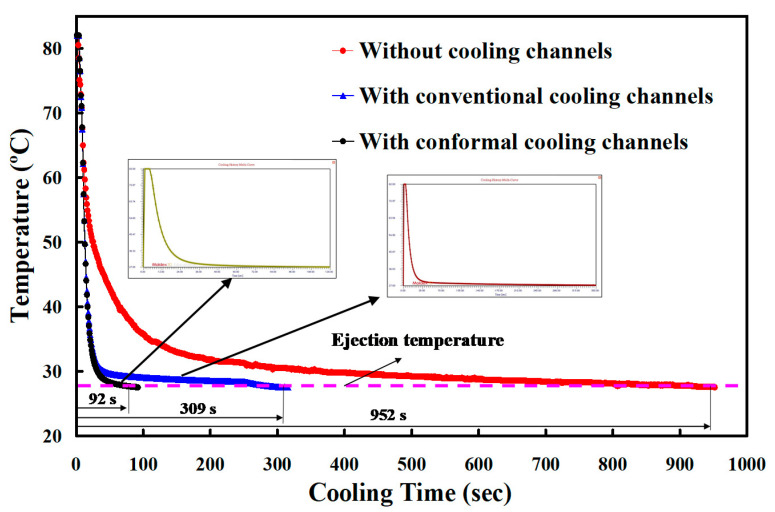
Cooling time for three injection molds.

The CCCs can be used to remove heat from areas where the conventional cooling channels cannot reach, especially for products with intricate geometry. In order to ensure the effectiveness of the cooling time for the injection mold with optimum CCCs, the cooling time of the three different injection molds was compared. The cooling time for the injection molds with conventional cooling channels and CCCs was obtained from the simulation, whereas the cooling time for the injection mold without cooling channels was obtained by the experiment. Figure 11 shows the cooling time for three injection molds. As can be seen, the cooling time for the injection molds without cooling channel, with conventional cooling channels, and CCCs was 952.1, 309.5, and 92.73 s, respectively. It is important to highlight that the cooling time for injection mold with optimum CCCs of about 90.26% was improved.

**Figure 12:**
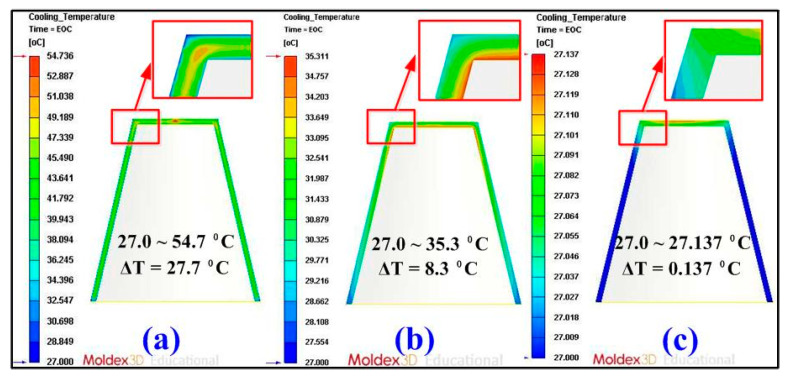
Numerical simulation results of part temperature difference for injection molds (**a**) without cooling channel, (**b**) with conventional cooling channels, and (**c**) with optimum conformal cooling channels (CCCs).

**Figure 13:**
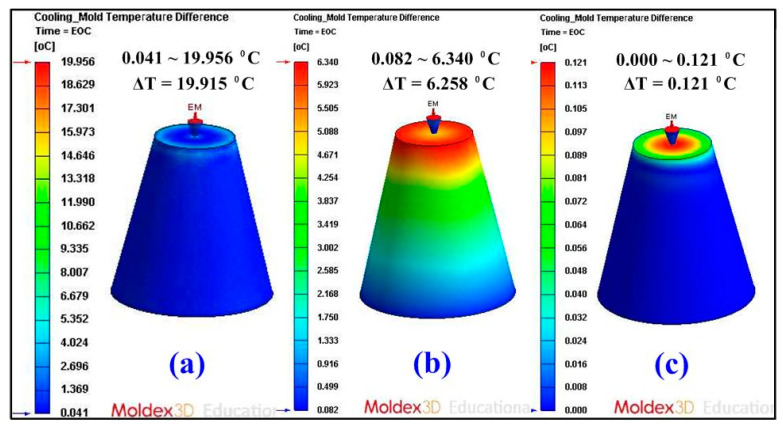
Numerical simulation results of mold surface temperature difference for injection molds (**a**) without cooling channel, (**b**) with conventional cooling channels, and (**c**) with optimum CCCs.

**Figure 14:**
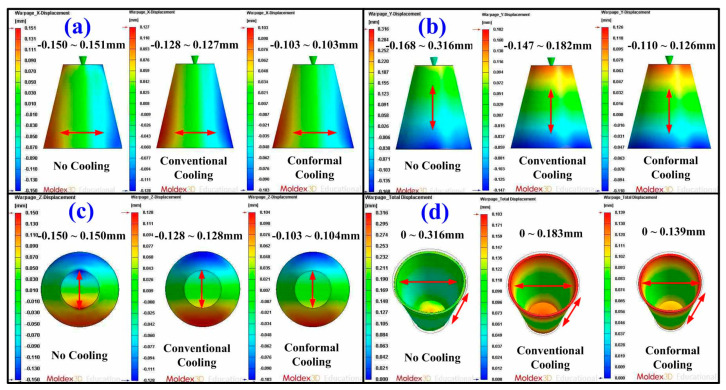
Numerical simulation results of total displacements in (**a**) x-direction, (**b**) y-direction, (**c**) z-direction, and (**d**) total directions.

Some defects such as weld lines, warpage [23], sink marks [24], shrinkage [25], and residual stress can be reduced if the part temperature difference or mold temperature difference was reduced. Figure 12 shows the numerical simulation results of part temperature difference for injection molds without cooling channel, with conventional cooling channels, and with optimum CCCs. Part temperature differences for injection molds without cooling channel, with conventional cooling channels, and with CCCs were 27.7, 8.3, and 0.137 °C, respectively. This means the part temperature difference for injection mold with optimum CCCs of about 99.51% can be reduced. Figure 13 shows the numerical simulation results of mold surface temperature difference for injection molds without cooling channel, with conventional cooling channels, and with optimum CCCs. Mold surface temperature differences for injection molds without cooling channel, with conventional cooling channels, and with optimum CCCs were 19.915, 6.258, and 0.121 °C, respectively. This means the mold temperature difference for an injection mold with optimum CCCs of about 99.39% can be reduced. Figure 14 shows the numerical simulation results of total displacements in the x-direction, y-direction, z-direction, and total directions. The total displacements for injection molds without cooling channel, with conventional cooling channels, and with optimum CCCs were 0.316, 0.183, and 0.139 mm, respectively. This means the total displacements for an injection mold with optimum CCCs of about 56.01% can be reduced. It was also observed that the improvement of the warpage was significant in all directions for an injection mold with optimum CCCs. Thus, an injection mold with optimum CCCs can improve product quality and some defects such as residual stress, weld lines, warpage, sink marks, and shrinkage can be reduced significantly.

**Figure 15:**
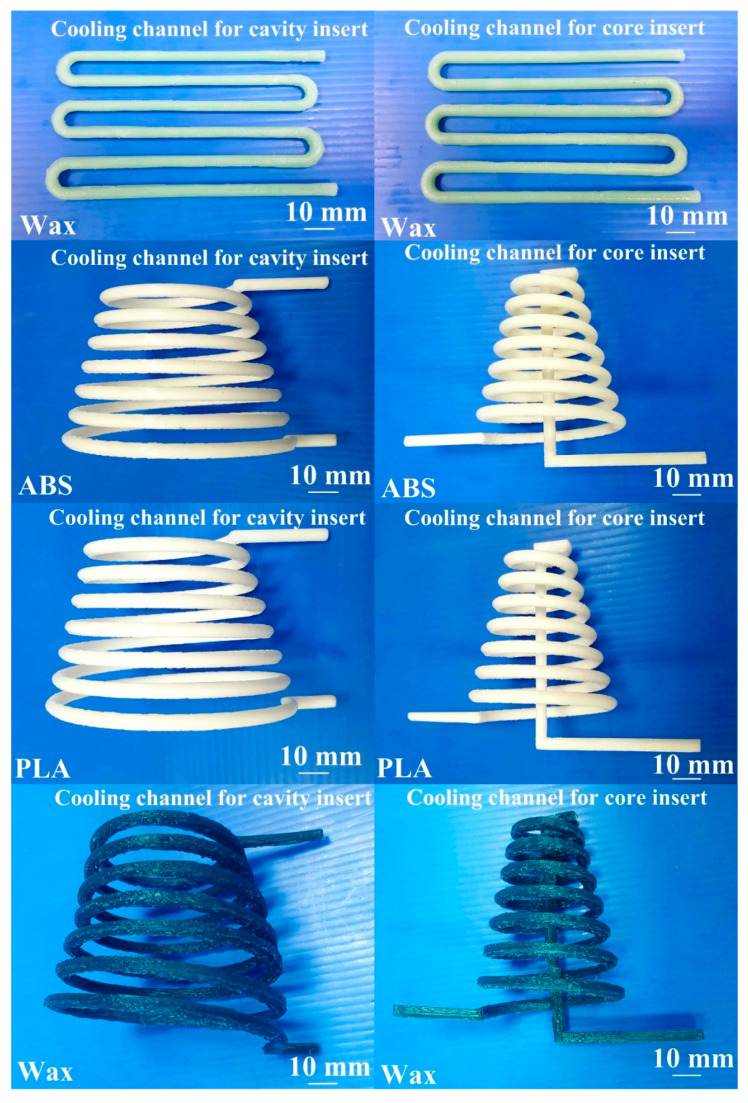
Photos of the conventional cooling channels and optimum CCCs.

**Figure 16:**
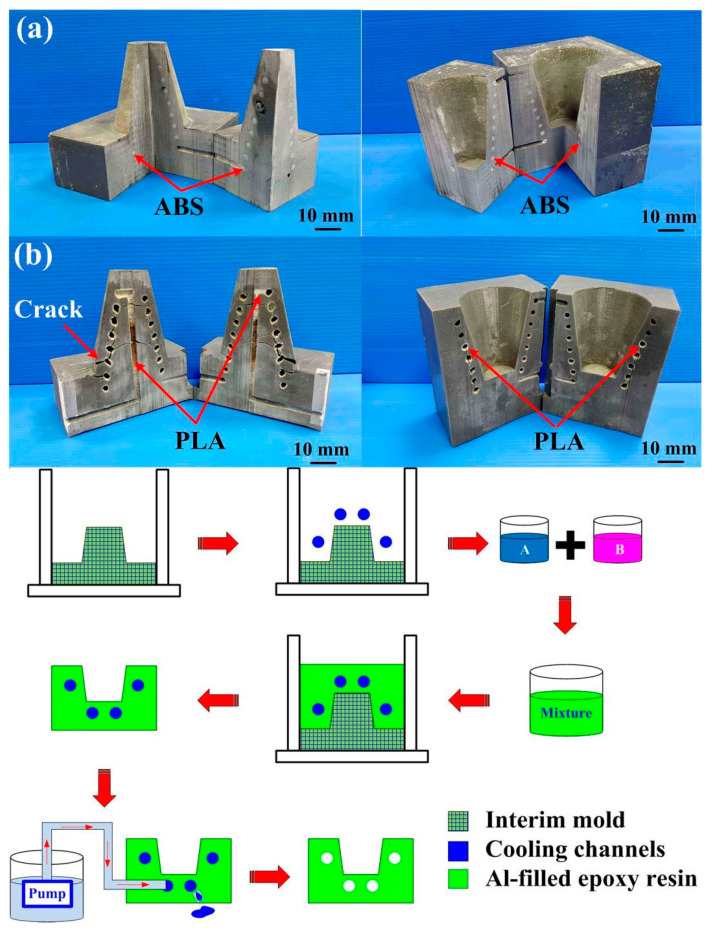
Failed injection molds with (**a**) ABS and (**b**) PLA cooling channels.

**Figure 17:**
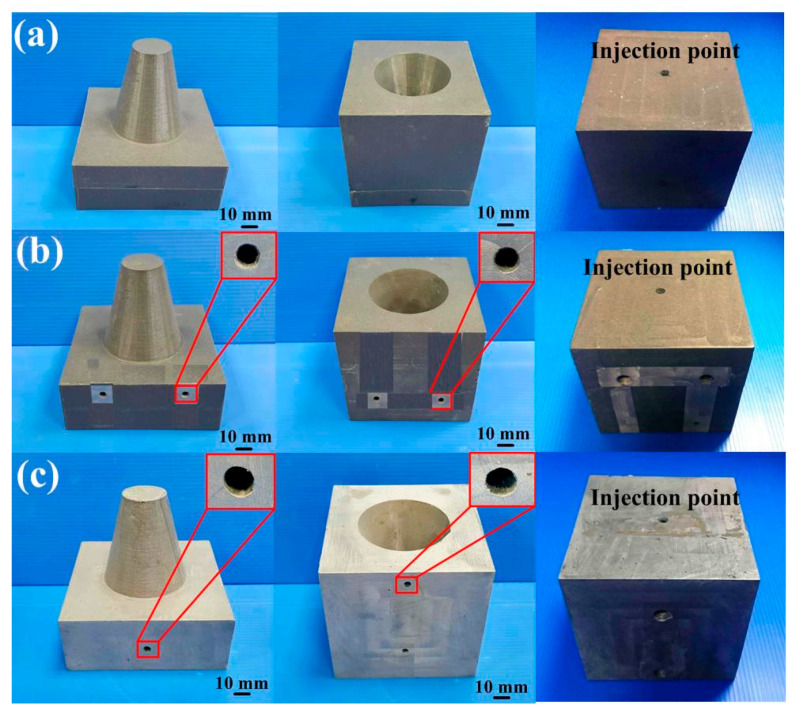
Accomplished injection molds (**a**) without cooling channel, (**b**) with conventional cooling channels, and (**c**) with optimum CCCs.

Figure 15 shows the photos of the conventional cooling channels and optimum CCCs. Three different materials were used to fabricate CCCs. Generally, the ABS CCCs inside the injection mold can be removed by flushing with acetone solvent since the ABS can be dissolved in acetone. The PLA CCCs inside the injection mold can be removed by flushing with industrial alcohol since the PLA can be dissolved in industrial alcohol solution. Figure 16 shows two failed injection molds. As can be seen, the cooling channels of both ABS and PLA plastics cannot be removed thoroughly from the inside of the injection molds completely. The possible reason is that the removing solution is difficult to chemically react with cooling channels inside the injection mold, especially in the cooling channels with small diameter and intricate geometries. In addition, it was found that the injection mold will crack during the removal process since the PLA cooling channels will expand caused by absorbing industrial alcohol during the removal process. Thus, it is interesting to note that the wax was strongly recommended as the material for making CCCs since the wax CCCs inside the injection mold can be removed completely and effectively by hot water of 95 °C. The removal results can be determined according the weights of the mold before and after removal process. Figure 17 shows the accomplished injection molds without cooling channel, with conventional cooling channels, and with optimum CCCs. The cooling channels were located at exact places. This result shows that an injection mold with sophisticated CCCs can be fabricated easily by RTT. Note that it is very difficult to fabricate these injection molds with intricate geometries using conventional machining [26]. In general, the surface quality of the mold fabricated by direct metal laser sintering, vacuum diffusion bonding, or electron beam melting was not good due to staircase effect [27]. A distinct advantage was found that no extensive machining was needed to obtain the required surface roughness and tolerance compared to an injection mold fabricated by direct metal laser sintering, vacuum diffusion bonding, or electron beam melting, since injection mold was fabricated by casting process [28,29,30,31].

**Figure 18:**
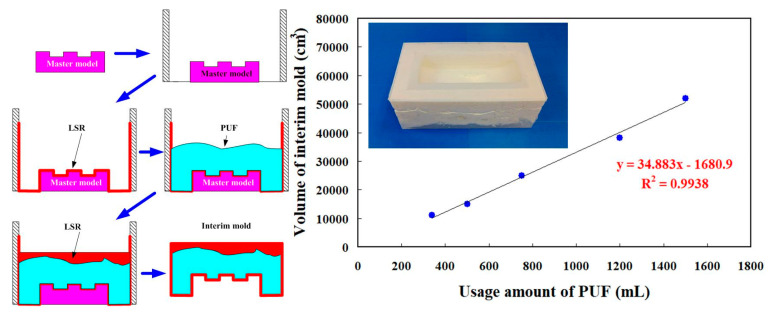
A innovative method for fabricating a large intermediary mold for large a wax injection mold.

Reduction of production cost is an important issue for a large injection mold. In general, an intermediary mold that is complementary in shape to the core or cavity inserts was commonly used for fabricating injection molds. However, the production costs of a large intermediary mold are expensive. To reduce the production costs, an innovative method using polyurethane foam as backing material to fabricate intermediary mold was proposed. Figure 18 shows an innovative method for fabricating a large intermediary mold for a large injection mold. It was found that *y* = 34.883*x*−1680.9 is a trend equation for predicting the usage amount of PUF. The *x* and *y* represent the usage amount of PUF and volume of intermediary mold, respectively. The larger the coefficient of determination (COD), the better the degree of accuracy of the trend equation [32,33]. Thus, the usage amount of PUF can be directly determined from the volume of intermediary mold since the COD was about 0.9938. The use of PUF in the manufacturing process of an intermediary mold can reduce the usage amount of new liquid silicone rubber (LSR) significantly, since the volume expansion of PUF is about 30 to 35 times. An intermediary mold was then fabricated by both PUF and LSR. The LSR was first used as surface material of the intermediary mold and the PUF was then used as the backing material. The LSR was applied uniformly to the surfaces of the master model and the sidewalls of the mold frame with the help of a brush. Finally, the backside of the intermediary mold was covered with LSR. Note that two distinct advantages including low investment and low running cost of ownership were obtained, which meet the needs of green manufacturing [34,35,36,37,38]. The developed intermediary mold had high load and supporting capacities, which can be used for manufacturing a large RT. The length, width, and height of a large intermediary mold were about 470, 270, and 180 mm, respectively. The costs of liquid silicone rubber and polyurethane foam are in new Taiwanese dollars (NTD) 735/kg and 300/kg, respectively. The production cost for the overall intermediate mold made of silicone rubber was about NTD 16,170. However, the production costs of the intermediary mold fabricated by the method proposed by this work were only about NTD 8100. It should be noted that production cost savings of about 50% for the intermediary mold fabricated by the innovative method was obtained. Accordingly, the innovative method has application potential for the large RT industry due to the production cost reduction increases with increasing the sizes of the intermediary mold.

**Figure 19:**
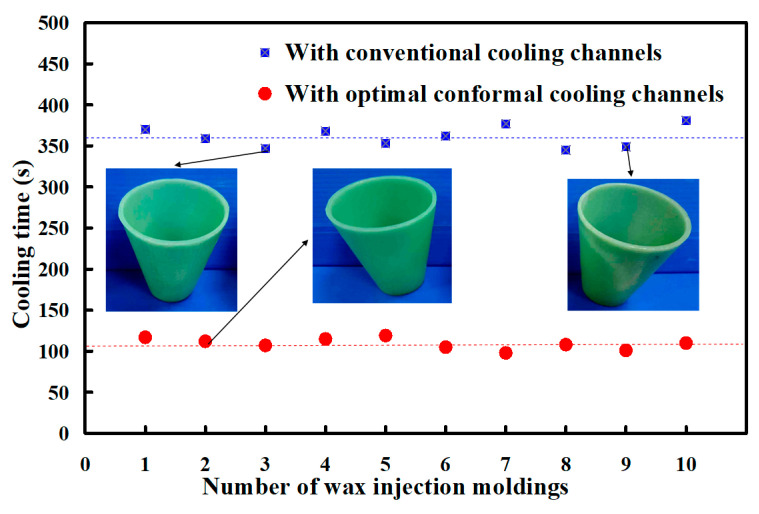
The cooling time for the ten injection moldings in the low-pressure wax injection molding process.

**Figure 20:**
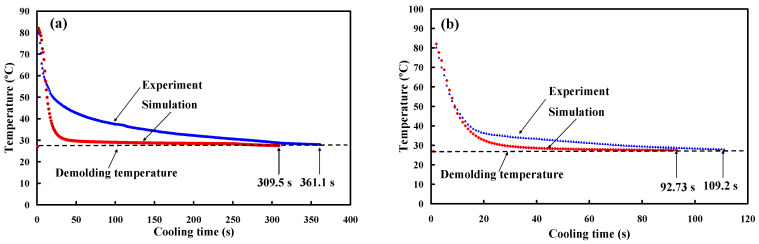
Comparison of simulation results with experimental results (**a**) an injection mold with conventional cooing channels and (**b**) an injection mold with optimum CCCs.

The cooling stage plays an important role in the cycle time of the injection molding process. To investigate the errors of the cooling time between experiment and simulation results, ten injection molding cycles were carried out. Figure 19 shows the cooling time for the ten injection moldings in the low-pressure wax injection molding process. As can be seen, the actual average cooling time for an injection mold with conventional cooling channels was about 361.1 s. The standard deviation was about 12.7 s. The actual average cooling time for an injection mold with optimum CCCs was about 109.2 s. The standard deviation was about 6.8 s. However, the actual cooling time for an injection mold without cooling channels was about 952.1 s. It is clear that the reduction in cooling time of about 89% was obtained. As can be seen, the low-volume production of wax patterns can be fabricated swiftly and inexpensively for investment casting of metal components with simple geometries [39,40]. The cooling time can be shortened for an injection mold with optimum, but the cooling efficiency is limited to the thermal conductivity of the mold materials. Therefore, it is necessary to change the mold materials if the cooling efficiency needs to be improved higher. To fulfill this requirement, a future work is required to develop new mold materials with higher thermal conductivity. In addition, the Taguchi method [41,42,43,44,45] was also recommended to reduce the number of simulations. As can be seen, the CCCs allowed the coolant to access all molded wax pattern locations uniformly. Therefore, the dimensional accuracy and surface appearance of the wax pattern were acceptable. Figure 20 shows the comparison of simulation results with experimental results for an injection mold with optimum conformal cooing channels and with conventional cooling channels. The average cooling time for an injection mold with conventional cooling channels was 361.1 s. The relative error was about 14.3% compared with that obtained by the simulation result of 309.5 s. In addition, the average cooling time for an injection mold with optimum CCCs was 109.2 s. The relative error was about 15.1% compared with that obtained from the simulation result of 92.73 s. The variation can be attributed to the difference between the experimental conditions and characteristic parameters such as molding material, mold materials, and responses of the injection machine used in the simulation software. These characteristic parameters [46,47,48] include thermal conductivity, melting point, specific gravity, linear shrinkage, viscosity, specific volume, heat capacity, viscoelasticity, density, elastic modulus, Poisson ratio, and coefficient of linear thermal expansion.

According to the results presented above, the findings in this study can be used as a reference to design CCCs for injection molds fabricated by RTT. Two distinct advantages were found. First, the production costs for a wax injection mold with sophisticated cooling channels is cost-effective because injections mold with sophisticated CCCs can be implemented economically and efficiently using both AM and RTT [49]. Second, the optimum conformal cooling channel with intricate shapes can be fabricated easily using AM technology [50,51]. However, the mechanical properties of the injection molds fabricated by Al-filled epoxy resins are inferior to metallic molds. Thus, some reinforcing fillers such as wollastonite, molybdenum disulfide [52], glass sphere, zirconia [53,54,55], silica sand, glass fibers , or silicon nitride [56,57,58] were recommended to add in the matrix materials. Additionally, metal AM technologies such as DB [59], SLS [60,61], SLM [62], electron beam melting, or direct metal laser sintering were recommended for manufacturing injection molds with optimum CCCs for mass production of wax patterns. The water was employed as the coolant. The alternative coolants such as cool air [63] or oil [64] can also be used in the experiment for investigating the difference in the cooling performance of an injection mold with optimum CCCs. Lastly, the molds or dies with CCCs fabricated by the proposed method can also be employed in metal injection molding [65], rotational molding [66], centrifugal molding, thermoforming [67], transfer molding [68], hot stamping [69], plastic injection molding [70,71], microinjection molding [72,73], or blow molding [74]. These issues are currently being investigated and the results will be presented in a later study.

## 4. Conclusions

According to practical experiences in injection molding, the cycle time is a very important parameter in injection molding because a longer cycle time stands for lower productivity due to cooling being a critical process. In general, the productivity is usually restrained by the cooling time because the cooling time takes most of the cycle time. This work explores a technology to develop an injection mold with high cooling performance by integrating molding simulation and rapid tooling technology. The findings are very practical and provide the greatest application potential in the precision mold or die industry, especially in the mold or die design stage. Based on the results discussed in this study, the following conclusions can be drawn:

1. An innovative method for fabricating a large intermediary mold for a large injection mold using polyurethane foam was proposed. A trend equation for predicting the usage amount of polyurethane foam was demonstrated. Production cost savings of about 50% was obtained.
2. The wax was found to be a good candidate as the material to fabricate CCCs because it can be removed completely and efficiently.
3. In an optimum conformal cooling channel design, the cooling channel diameter, center distance between cooling channels, and center distance with respect to mold cavity were 4, 6, and 8 mm, respectively. A reduction in the cooling time of about 89% can be obtained when the optimum CCCs were used in the injection mold. The variation of the results between the experiment and the simulation was approximately 15.1%.
4. The benefits of the simulation in the cooling time, part temperature difference, mold surface temperature difference, and product total deformation were 47.28, 72.01, 72.9, and 38.22%, respectively.

## Figures and Tables

**Table 1:** Forty-five cases of conformal cooling channel design.

Case Number	d (mm)	P (mm) P = 1.5 d to 2 d	L (mm) L = 2 d to 3 d	Case Number	d (mm)	P (mm) P = 1.5 d to 2 d	L (mm) L = 2 d to 3 d	Case Number	d (mm)	P (mm) P = 1.5 d to 2 d	L (mm) L = 2 d to 3 d
1	4	6	8	16	5	10	10	31	7	11.9	14
2	4	6	10	17	5	10	12.5	32	7	11.9	17.5
3	4	6	12	18	5	10	15	33	7	11.9	21
4	4	6.8	8	19	6	9	12	34	7	14	14
5	4	6.8	10	20	6	9	15	35	7	14	17.5
6	4	6.8	12	21	6	9	18	36	7	14	21
7	4	8	8	22	6	10.2	12	37	8	12	16
8	4	8	10	23	6	10.2	15	38	8	12	20
9	4	8	12	24	6	10.2	18	39	8	12	24
10	5	7.5	10	25	6	12	12	40	8	13.6	16
11	5	7.5	12.5	26	6	12	15	41	8	13.6	20
12	5	7.5	15	27	6	12	18	42	8	13.6	24
13	5	8.5	10	28	7	10.5	14	43	8	16	16
14	5	8.5	12.5	29	7	10.5	17.5	44	8	16	20
15	5	8.5	15	30	7	10.5	21	45	8	16	24

**Table 2:** Boundary and initial conditions for numerical simulation.

Parameters	Value
Density of injection mold (g/cm^3^)	1.95
Heat capacity of injection mold (cal/g °C)	1.97
Thermal conductivity of injection mold (W/m·K)	10.82
Elastic modulus of injection mold (GPa)	2.54
Poisson ratio of injection mold	0.17
Melting point of the molding material (°C)	85
Specific gravity of the molding material	0.96
Linear shrinkage of the molding material (%)	0.9–1.0
Poisson ratioof the molding material	0.17
Part thickness (mm)	2
Filling time (s)	4.3
Injection pressure (MPa)	0.18
Coolant flow rate (L/min)	10
Injection temperature (°C)	82
Mold temperature (°C)	27
Coolant temperature (°C)	27
Ejection temperature (°C)	27

**Table 3:** Simulation results of the forty-five cases.

Case Number	Cooling Time (s)	Part Temperature Difference (°C)	Mold Surface Temperature Difference (°C)	Total Displacement (mm)	Case Number	Cooling Time (s)	Part Temperature Difference (°C)	Mold Surface Temperature Difference (°C)	Total Displacement (mm)
1	92.73	0.137	0.121	0.139	24	164.52	0.381	0.233	0.14
2	94.75	0.142	0.125	0.139	25	171.43	0.298	0.189	0.14
3	101.94	0.166	0.136	0.139	26	177.76	0.353	0.198	0.14
4	104.99	0.151	0.133	0.14	27	162.49	0.408	0.23	0.141
5	109.01	0.165	0.129	0.141	28	168.53	0.3	0.195	0.14
6	114.12	0.179	0.131	0.139	29	174.75	0.353	0.218	0.141
7	123.18	0.173	0.123	0.139	30	193.46	0.405	0.244	0.14
8	129.31	0.188	0.119	0.138	31	201.25	0.446	0.298	0.14
9	134.44	0.203	0.117	0.138	32	206.98	0.506	0.335	0.141
10	106.8	0.123	0.084	0.086	33	224.02	0.554	0.356	0.141
11	124.84	0.188	0.118	0.135	34	232.76	0.758	0.546	0.142
12	131.9	0.199	0.122	0.138	35	245.5	0.811	0.576	0.142
13	133.91	0.188	0.129	0.138	36	252.14	0.853	0.593	0.142
14	139.94	0.19	0.126	0.139	37	232.32	0.53	0.527	0.141
15	145.13	0.232	0.131	0.139	38	237.45	0.671	0.577	0.141
16	155.25	0.276	0.137	0.14	39	240.64	0.706	0.585	0.141
17	167.4	0.297	0.134	0.14	40	244.4	0.898	0.679	0.142
18	172.5	0.342	0.141	0.14	41	250.03	0.964	0.711	0.143
19	151.96	0.206	0.191	0.139	42	256.5	0.926	0.721	0.145
20	157.08	0.243	0.196	0.139	43	260.42	1.181	0.883	0.145
21	160.17	0.302	0.173	0.139	44	262.22	1.25	0.923	0.146
22	167.19	0.314	0.175	0.14	45	264.05	1.279	0.937	0.146
23	173.3	0.334	0.203	0.14					

## Data Availability

Data openly available in a public repository that issues datasets with DOIs.
